# Sub-lethal imidacloprid exposure leads to presynaptic and postsynaptic alterations

**DOI:** 10.21203/rs.3.rs-7428768/v1

**Published:** 2025-10-08

**Authors:** Katya Tjahaja, Emma Stoner, Akari Miura, Cole Damon, Jake Romley Murias, Denise B. Flaherty, Scott E. Dobrin

**Affiliations:** Eckerd College; Eckerd College; Eckerd College; Eckerd College; Eckerd College; Eckerd College; Eckerd College

## Abstract

Pesticides are commonly used in agriculture to mitigate a wide array of pests. Imidacloprid, the most frequently used neonicotinoid pesticide, has been reported to affect cholinergic signaling of off-target organisms, such as honey bees and nematodes. Taking comparative neuroscience approaches, we exposed both organisms to field-relevant sub-lethal doses of imidacloprid. In the honey bee brain, we examined microglomeruli (synaptic structures of the mushroom bodies) using synapsin immunostaining. There was a decreased density of microglomeruli following one week of imidacloprid exposure. Furthermore, to take a separate look at either side of cholinergic synapse, we used two distinct strains of transgenic nematodes labeling either presynaptic or postsynaptic cholinergic structures. Following 48 hours of exposure, we found decreased expression in both strains. These results indicate that concentrations of imidacloprid commonly occurring in the field alter the nervous systems of off-target organisms.

## 1. Introduction

Global pesticide usage is over 3.7 million metric tons each year^1^. Excessive pesticide exposure has detrimental effects on biodiversity and population of off-target organisms, including birds, aquatic organisms, and insects^2^. Neonicotinoid pesticides are designed to target specific pests as an agonist of nicotinic acetylcholine receptors^3^. These receptors impact both cellular physiology and behavior, evidenced by alteration of resting membrane potential, synaptic transmission, and fast excitatory transmission, as well as alterations in learning and memory of both humans and invertebrate animals^4^.

Imidacloprid, a neonicotinoid pesticide, is commonly used to control the spread of barley yellow dwarf luteovirus (BYDV) in wheats and oats, allowing agriculturists to maintain a high yield of cereal grains^5^. The water-soluble property of imidacloprid allows a systemic response through absorption via plant roots and leaves and transport throughout their structure. Though imidacloprid has been shown to protect crops and maintain high yield, the negative impacts on off-target organisms, like pollinators, including honey bees, or beneficial soil decomposer insects, cannot be ignored^6^. Imidacloprid is effective in mitigating various pest species of different orders^7^. Insect nicotinic receptors are susceptible to imidacloprid due to increased receptor binding^8^.

Various off-target species are negatively impacted following imidacloprid exposure, either by soil run-off or through contact contamination. In rice paddy irrigation ponds in Japan, which typically contain high taxonomic richness, a 15 % reduction of invertebrate populations, including 77 % of large insects, was found following imidacloprid exposure^9^. Mortality, abundance, biomass, behavior, reproduction, biochemical biomarkers, growth, richness and diversity, and structural changes also are negatively impacted in terrestrial vertebrates following imidacloprid exposure^10^. The range of ecosystems negatively impacted by imidacloprid is evidenced by toxicity being reported in crustaceans, marine invertebrates, and land-dwelling insects^2^.

This report explores how imidacloprid in the environment may alter the brain structure of off-target organisms. Honey bees (*Apis mellifera*) pollinate more than $15 billion worth of crops annually, including more than 130 types of fruits, nuts, and vegetables^11^. The nematode worm, *C. elegans*, are critical decomposers in soil habitats used as an indicator of crop and soil health^12,13^. Both are also animal models which offer advantages to study distinct aspects of the neurobiological effects of imidacloprid.

Due to their complex social behaviors and impressive navigational talents, the cognitive abilities of honey bees have been studied for decades ^14,15^. As technologies progressed, the brain structure, including the general architecture and overall molecular layout, began to be outlined^16,17^. Today we know that the ~1 mm^3^ honey bee brain consists of an estimated 960,000 neurons^18^ which utilize acetylcholine as the primary excitatory neurotransmitter^19,20^. *C. elegans*, alternatively, are a valuable model of human diseases because they have 83% genetic conservation to humans while containing a defined number and distribution of neurons^21^. Moreover, the genetic tractability of *C. elegans* permitted mapping of the connections between all 302 somatic neurons in the nervous system^22,23^, including the 159 cholinergic neurons. Cholinergic pathways in *C. elegans* perform many roles and are composed of sensory neurons (simple and polymodal), interneurons, and motor, with motor neurons being the most predominant type^24^.

While each animal is capable of learning, the brain areas responsible for doing so are distinct. The protocerebrum of the honey bee brain houses the so-called mushroom bodies, paired nerve plexuses named after their visual similarity to a cap and stalk of a mushroom^25–27^. The intrinsic neurons of the mushroom body, Kenyon cells, receive input from olfactory, visual, and gustatory projection neurons^28^. It is these synapses that are altered during normal development^29,30^, with foraging experience^29,31–33^, following associative learning^32–35^, and as a result of pesticide exposure^36–39^. The nerve ring that circulates the pharyngeal bulbs of *C. elegans* is analogous to a brain structure in that it is the site of integration and processing of sensory and motor information. Nicotinic acetylcholine receptors, and cholinergic vesicle-filling transporters, are found not only in the pharyngeal nerve ring, but also in the ventral nerve cord, along the body-wall muscles and uterine muscles^24,40,41^. These cholinergic pathways are responsible for locomotion, and egg laying behavior, repulsion to odor sensory cues and have a role in sex development as well^25,42,43^.

Exposure to imidacloprid alters both honey bee and *C. elegans* biology. Changes in honey bee foraging behavior^44^, neuronal activation^45^, and learning^46–51^ have been reported. Feeding honey bee larvae imidacloprid-contaminated food for 4 days results in decreased size and density of the mushroom body synaptic structures, microglomeruli^36^. Microglomeruli consist of the terminals of projection neurons surrounding the spines of Kenyon cell dendrites^52^ which can be imaged using immunohistochemical techniques (for review^27^). *C. elegans* exposed to a commercial product containing imidacloprid demonstrate delayed growth, disrupted locomotion, and decreased fertility^42^. As with honey bees, morphological abnormalities are reported in the cholinergic neurons^42^. Another study found that imidacloprid reduces *C. elegans* body length, maturation rates, and the number of offspring for up to three generations after initial exposure^53^.

In this study, we focused on the responses of synaptic connections to field-relevant, sub-lethal doses of imidacloprid delivered to adult honey bees and *C. elegans* over an extended period of time. Due to differences in natural exposure, the routes, concentration, and duration of imidacloprid administration used for each model was controlled. Honey bee brains were processed using immunohistochemistry to measure differences in the density of the synaptic structures of the mushroom body, the microglomeruli. Whereas *C. elegans* expressing transgenic markers specifically labeling vesicular acetylcholine transporters or nicotinic acetylcholine receptors allowed distinct investigation of presynaptic or postsynaptic involvement. By examining the cellular neuroanatomical changes induced by low imidacloprid exposure on two important off-target species a better picture of pesticide impacts was ascertained.

## 2. METHODS

### 2.1. Honey bee (*A. mellifera*)

#### *A. mellifera* Maintenance and Imidacloprid Exposure

Honey bees were maintained in the Eckerd College Apiary in St. Petersburg, Florida. Frames of brood were removed from the hive and maintained in an incubator at 33 ℃ and 60 % relative humidity overnight. At the start of each experimental replicate, twenty newly emerged bees (1–12 hours post larval hatching) were placed in 90 cm × 90 cm × 9 cm custom clear Plexiglass boxes. Each cage was maintained in an incubator at 30 ℃ and 60 % relative humidity. Two 1.5 mL microcentrifuge tubes filled with 30 % sucrose were provided in every box so honey bees could feed *ad libitum*. Sucrose was made weekly and stored at 4 ℃. Fresh tubes of sucrose were supplied daily to prevent ethanol fermentation. Dead bees were removed from each cage daily under red light and mortality was tracked. Newly emerged honey bees were caged for seven days and fed sugar containing concentrations of imidacloprid at levels found in the nectar and pollen of returning foragers^54^.

Field-relevant doses of imidacloprid are informed by the previous literature examining the concentrations detected in pollen, nectar and bee bread from returning foragers (Mullin et al., 2010). Imidacloprid was presented at three concentrations: control (0 ppb), low (10 ppb), and high (20 ppb) based on research by a previous report^55^ examining sub-lethal dosages of imidacloprid on honey bees. Imidacloprid was dissolved in DMSO to make a stock solution of 400 ng/μl. This was diluted in 30 % sucrose to make the appropriate dosed-feeding solutions. Control tubes had an equal concentration of DMSO as the high tubes. Sucrose solutions were changed daily to prevent ethanol fermentation. Honey bees which do not obtain a carbohydrate source for longer than 48 hours will not survive; thus, all bees are known to be exposed to pesticide after 7 days in the cage.

#### Immunohistochemical imaging

After seven days of exposure to imidacloprid, cages were placed at 4 ℃ until the bees were still. Individual honey bees were removed from the cages and dismembered prior to brain dissection under a microscope in phosphate buffered saline (PBS; Millipore Sigma, AL). Brains were fixed in 4 % paraformaldehyde (Electron Microscopy Sciences, PA) overnight at 4 ℃. The brains were embedded in 7 % agarose (ThermoFisher, MA) and longitudinally sectioned at 100 μm on a Compresstome vibrating microtome (Precisionary Instruments, Massachusetts). Individual sections were placed (maintaining order) in wells of a 96-well plate. Mushroom body synaptic structures can be visualized via colabeling of presynaptic SYNORF1 and postsynaptic actin markers^27,52,56^. Sections were washed with 0.1 % Triton X-100 in PBS (Sigma Aldrich, MA), blocked with 0.25 % PBST-Bovine Serum Albumin (BSA) in 2 % Normal Goat Serum (NGS; Jackson Immunoresearch laboratory, PA) and then treated with 7.5 % 3C11 (anti-SYNORF1), which was deposited to the DSHB by Buchner [DSHB Hybridoma Product 3C11 (anti-SYNORF1), Iowa], for three days at 4 ℃. After three days, brains were washed in 0.25 % PBST-BSA and treated with 2 % secondary antibodies. Goat anti-Mouse-Fluorescent-Cy5 (Jackson ImmunoResearch Inc, Pennsylvania) for four (4) days. After four days, brains were washed in PBS and underwent autofluorescence quenching with the Vector TrueView Kit (SP-8400; Vector Labs, California) according to manufacturer’s instructions prior to mounting on a 1 mm × 3 mm × 1 mm microscope slides (Mercedes Medical, FL) with 22 mm × 22 mm × 2 mm coverslips (Amscope, California) using Vectashield hardset mounting media with DAPI to stain chromatin in the nuclei of cells.

#### *A. mellifera* Imaging, Analysis and Statistics

**Figure 1:**
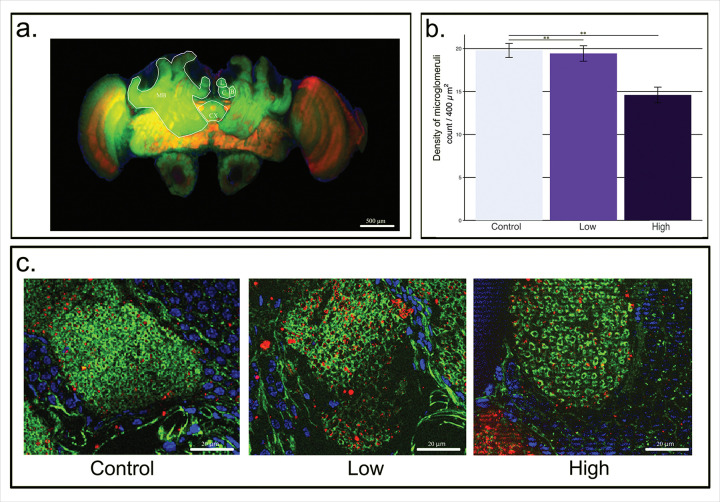
Imidacloprid treatment decreases microglomeruli density of the honey bee. **a.** A representative honey bee whole brain fluorescence micrograph at the level for analysis. One of the paired mushroom bodies (MB) is outlined and the subdivisions of the calyx of the other mushroom body, the lip (L), collar (C), and basal ring (B), are separately outlined. Either of the medial lips was chosen at random for imaging only in sections with a clearly identifiable central complex (CX). Scale bar = 100 μm **b.** Comparison of density of microglomeruli across different treatments: control (0 ppb), low (10 ppb), and high (20 ppb). A significant difference was found between all groups (F_2,15_ = 8.694, p = 0.003). Post-hoc, pairwise comparisons indicate significant differences between high and control (p = 0.003, **) as well as high and low groups (p = 0.008). No statistical difference was found between control and low groups (p = 0.953). Error bars indicate standard error of the mean. **c.** Representative fluorescence micrographs of a lip from each treatment imaged at 1000x final magnification. Scale bar = 20 μm. For both a. and c., Green represents actin (postsynaptic), red represents SYNAPSIN (presynaptic ), and blue represents nucleic acids (cell nuclei).

Brain sections were imaged using the M2 Zeiss Axio Imager. Sections were screened at 200 X and then 400 X total magnification to identify only the regions of the brain in which the central complex was clearly visible (Fig. 1). This ensured the same depth of mushroom body, in the center of the brain, was imaged. Each mushroom body has 2 calyxes, each with 2 lip regions. A single of the medial lips was imaged for analysis. Using Zen 2.6 Blue (Zeiss), an image at 400 X was taken to orient the analysis before capturing another at 1000 X (NA 1.4) image under oil immersion. Three channels (eGFP (488ex/509em): actin; DAPI(358ex/461em): nuclei; and Cy5 (647ex/665em): synapsin) were taken with an apotome for 10 – 20 μm of depth at a 0.25 μm z-step. Images were deconvolved prior to identifying a single image to export from the stack for analysis.

Images were processed in ImageJ by creation of TIFF images. Two circular selections of 22 μm diameter (400 μm^2^ area) circles were then analyzed for the amount of boutons (postsynaptic ring around presynaptic center) per circle^57^. Each lip analyzed had the density determined using one circle added to the upper left and another lower right, making sure to avoid the outermost layer of cells. The average value of both circles was considered a single sample. Statistical analyses were conducted using one-way ANOVA (p < 0.05) followed by Tukey post-hoc analysis. Results are presented as mean ± SD.

### 2.2. Nematode (*C. elegans*)

#### *C. elegans* Maintenance and Imidacloprid Exposure

Strains N2, CZ631 and LX929 were ordered from the *Caenorhabditis Genetics Center* (CGC) of the University of Minnesota. The methods for maintenance of *C. elegans* followed Epstein and Shakes^58^. Agar plates were made with sterile nematode growth media and kept in a 19.5 ℃ incubator. A supply of food was provided on the plates through seeding with the OP50 strain of *E. coli*. Transgenic *C. elegans* synchronized in age were grown for two days on plates containing imidacloprid at levels reported in soil^59^. Differences in the duration of exposure reflect the differences in expected lifespan of honey bees (~60 days) and *C. elegans* (18–20 days).

Daily monitoring of the plates was necessary to avoid contamination and starvation. Moreover, “chunking” by removing a populated portion of the agar and flipping it onto a fresh NGM plate with a sterile spatula was necessary to ensure starvation did not occur. Another method, “picking”, was implemented using a sterile platinum wire to individually remove and displace worms from one plate to another. In order to maintain sterility, and provide a food source that would not risk metabolizing imidacloprid, OP50 cultures were paraformaldehyde (PFA) treated for experimental treatment plates. The OP50 was grown in Luria Broth (LB) overnight at 37 ℃ with aeration at 200 rpm on a shaker. The paraformaldehyde treatment of 0.25 % for 90 minutes also allowed for the amount of food to be limited. The OP50 was then centrifuged at 3000 × g for 20 minutes. The supernatant was aspirated, and the pellet was resuspended then washed with sterile L-broth three times in order to ensure a homogeneous mixture free of PFA. The resultant pellet was resuspended to a final concentration of 0.1 g/mL of L-Broth. For the 35mm test plates, 10uL of PFA-treated OP50 was pipetted onto the plate and allowed to dry overnight.

In preparation for experiments, two types of embryo preparations were performed subsequent to one another in order to synchronize the developmental age of the animals. The first embryo preparations were Clorox/Hypochlorite Embryo Preparations in which plates with gravid (heavily pregnant) hermaphrodites and a large number of eggs were present^58^. Briefly, plates were “power-washed” with sterile M9 buffer and the liquid was transferred into a sterile 15mL conical tube in order to be centrifuged. Supernatants were aspirated and the pellet was exposed to hypochlorite, shaken vigorously, and washed with M9 four times. Embryos were then transferred into new NGM/OP50 100 mm plates in tiny aliquots and left to dry for 10 minutes before being stored in 19.5 ℃ incubators. The Hypochlorite Preparation was useful to disinfect the strains in addition to generally synchronizing the ages of the specimens.

In order to further synchronize the ages of nematodes, a picked population of gravid adults method was used. 96 hours post-Hypochlorite Preparation, 50 gravid hermaphrodites were placed on a new 100 mm NGM/OP50 plate and left to lay their embryos for two to three hours. Another two to three hours later, all hermaphrodites were removed from the plate. This method has been demonstrated as the most effective mechanism to truly synchronize the ages of animals^60^.

Three days (72 hours) after the population picked preparation, twenty worms were transferred to a new experimental plate of NGM/OP50/FuDR. FuDR (floxuridine) was present to prevent further egg-hatching. The new plates had one of three concentrations of imidacloprid, control (0 μg/mL), low (1 μg/mL), and high (10 μg/mL) dosed at 100μL per plate. These doses were distinct from the honey bee exposure concentrations because we focused on soil concentrations of imidacloprid to simulate the natural environment of the nematode^59^. The imidacloprid stock of 400 ng/μl was dissolved in DMSO, then diluted in water and directly pipetted onto plates, being left to dry for 5 minutes. After the imidacloprid dried, twenty adult worms were picked onto the plate.

#### *C. elegans* Mortality and Motility Assays

Two days (48 hours) after exposure to imidacloprid (day 5 post egg-lay animals), specimens were evaluated for mortality. As a part of the mortality assay, worms were also examined for paralysis. In *C. elegans*, a worm may be moving only their head part and thus, are scored as alive, but paralyzed. Full-body motion, as signified by bending of the body, also determines whether the animal is paralyzed or not. The tap reflex was investigated through a tap-withdrawal protocol that measures the responsiveness to a single tap, which vibrates the organism’s environment and will cause them to swim backwards in the opposite direction^56^. Positive tap results, shown by the described reversal behavior, indicate the worm can both sense and respond to environmental stimuli. A negative result, in which the worm stays still or continues in the direction of stimuli indicating a lack of response to stimuli.

Standard motility measures in beats per minute (thrashing assay) were also taken to determine how imidacloprid affects overall motor function. To do this, animals were taken from each of the plates and were picked off the plate onto a depression slide with 20 μL of M9 buffer. The specimens were allowed to adjust to the new liquid environment for 30 seconds. The nematodes’ dorsal and ventral muscles coordinated movement to create the characteristic sinusoidal patterns of movement, which are called bends. After 30 seconds, each body bend, marked by the curving of their entire body and returning to its original position, was counted for 30 seconds. The number of body bends was multiplied by two in order to determine beats per minute.

#### *C. elegans* Imaging, Analysis and Statistics

In order to image fluorescent neurons, *C. elegans* specimens that have completed the motility assay were transferred to a new imaging slide with 13 μL of M9 and 2 μL of 0.1M sodium azide which renders them paralyzed after 15 seconds. After the specimen was paralyzed, a coverslip was placed.

Using the Zeiss Axio Imager, other strains were imaged using eGFP at 400 X focusing on the nerve ring of the animal. After imaging, the photographs were analyzed with ImageJ to calculate total body volume, fluorescence, and nerve ring fluorescence. Analysis entailed using the polygon tool to trace over the entire body of the animal and measuring mean fluorescence. The nerve ring was measured at a set scale of 8.811 microns and a circle of 39 μm in diameter was drawn to encircle the nerve ring. Then, mean fluorescence was measured. Statistical analyses were conducted via one-way ANOVA (p < 0.05) followed by Tukey post-hoc analysis. Results are presented as mean ± SD.Images were then adjusted for printing using Photoshop by adjusting the Image Levels to range from 0 to 150 uniformly for both strains of total body nematode, as well as nerve rings.

## 3. RESULTS

### Honey bee microglomeruli density decreases with imidacloprid treatment.

Imidacloprid did not affect honey bee mortality during 7-day exposure to high or low doses. To investigate the effects of imidacloprid on synaptic complexes in the honey bee brain, we fed honey bees with sugar containing imidacloprid before dissecting their brains for subsequent immunohistochemistry (Fig. 1a). In the upper left, a single representative section of the honey bee brain is depicted. Highlighted is a mushroom body, each of the 2 potential lips for analysis (one used per brain), and the central complex. Note the representative image of the microglomeruli from each treatment (Fig. b) and the graphical summary of the data (Fig. 1c). The high treatment group had significantly lower density of microglomeruli than either the low or control groups (F_2,15_ = 8.694, p = 0.003).

### Imidacloprid decreases postsynaptic AChR expression in the nematode

**Figure 2:**
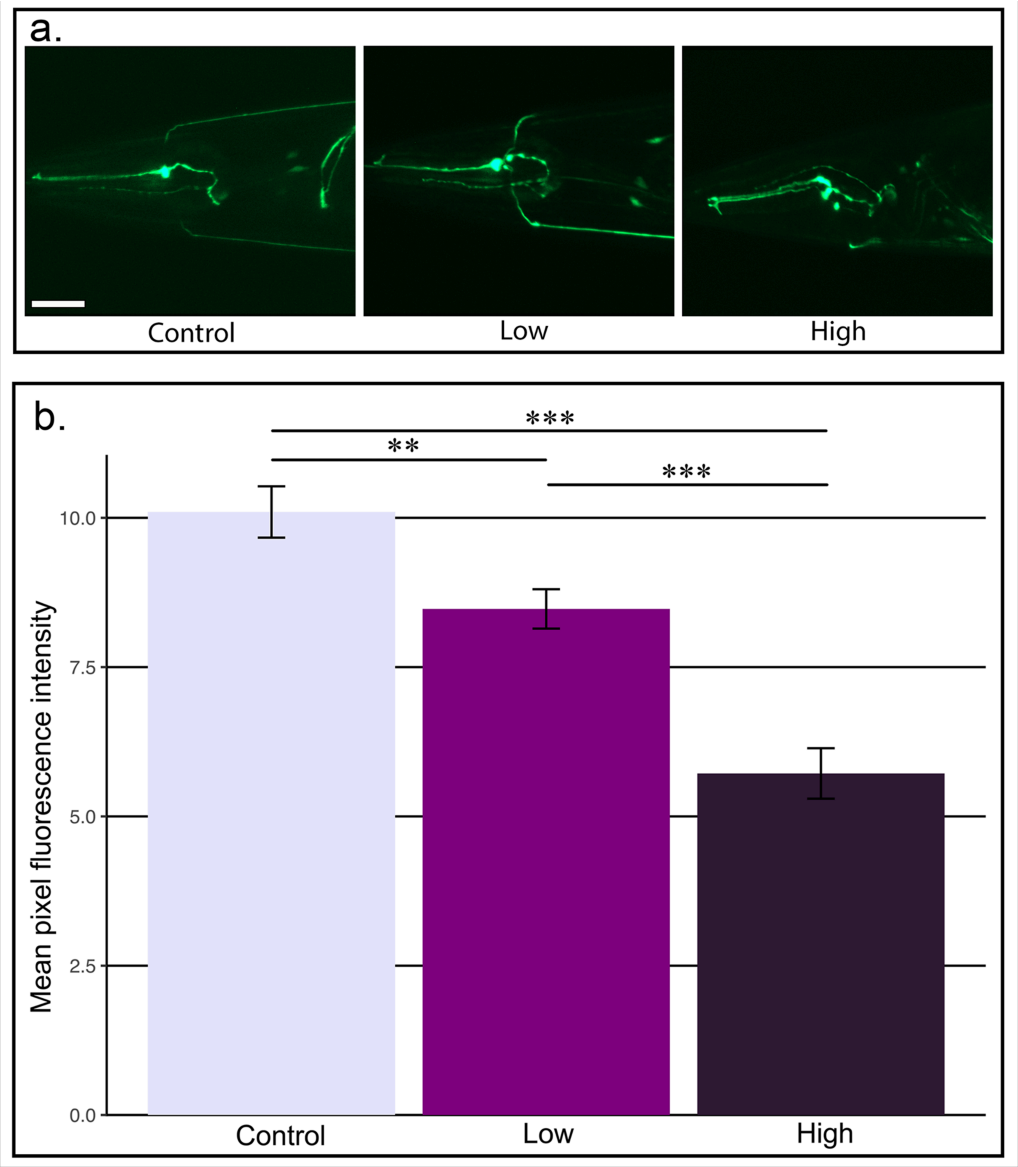
Postsynaptic receptors decrease with increasing doses of imidacloprid. **a.** Representative fluorescence micrographs of nerve rings from CZ631 animals taken at 400x total magnification. Animals were treated for 3 days as control (0 μg/mL), low (1 μg/mL) or high (10 μg/mL) groups. Scale bar = 20μm. **b.** Fluorescence intensity measurements indicate a significant difference between all groups (F_2,40_ = 30.14, p < 0.001). Post-hoc, pairwise comparisons indicate significant differences between all groups: high vs control (p < 0.001, ***), high vs. low groups (p < 0.001), and control vs low (p = 0.015, **). Error bars indicate standard error of the mean.

Microglomeruli are complexes of pre-and postsynaptic structures. In order to explore the mechanism of these changes, we treated strains of *C. elegans* characterized by expressing markers of specific synaptic structures. The *C. elegans* strain CZ631, which has fluorescently-labeled postsynaptic AChR transgene, was utilized to quantify changes at the postsynaptic membrane. In this way, modulation of potential imidacloprid-receptor interactions could be measured. Developmentally synchronized CZ631 *C. elegans* were examined after 48 hours of imidacloprid exposure (n=20/plate in triplicate). Mean pixel fluorescence was measured to determine the expression of postsynaptic AChR within each individual. Increasing doses of imidacloprid cause decreased postsynaptic markers in their nerve ring (F_2,40_ = 30.14, p < 0.001; Control vs Low: p = 0.015; Control vs High: p < 0.001; High vs Low: p < 0.001; Fig. 2a & b).

### Imidacloprid decreases presynaptic vesicular transporter expression

**Figure 3:**
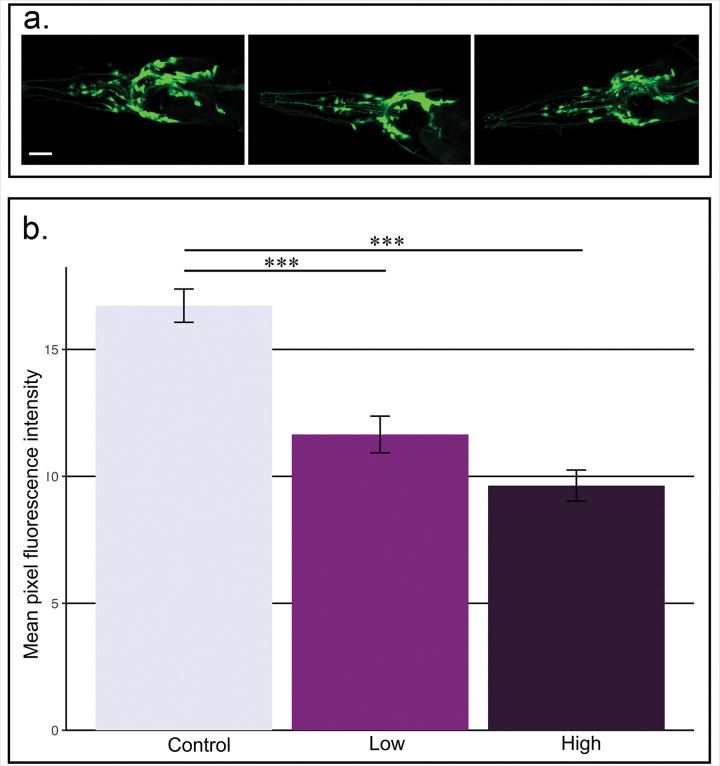
Imidacloprid treatment decreases expression of presynaptic vesicular acetylcholine receptors. **a.** Representative fluorescence micrographs of nerve rings from LX929 animals taken at 400x total magnification. Animals were treated for 3 days as control (0 μg/mL), low (1 μg/mL) or high (10 μg/mL) groups. Scale bar = 20μm. **b.** Fluorescence intensity measurements indicate a significant difference between all groups (F_2,61_ = 31.29, p < 0.001). Post-hoc, pairwise comparisons indicate significant differences between control and high (p < 0.001, ***) as well as control and low groups (p < 0.001). No statistical difference was found between high and low groups (p = 0.091). Error bars indicate standard error of the mean.

While imidacloprid directly binds to acetylcholine receptors, a neuronal synapse acts as a single structure. Therefore, we also wanted to explore what presynaptic changes might be occurring. We used LX929, a *C. elegans* strain which has the presynaptic vesicular transporter transgenically labeled, to observe changes of expression. Similar to our findings of the postsynaptic strain, we found that imidacloprid causes decreased presynaptic vesicular transporter expression (F_2,61_ = 31.29, p < 0.001; Control vs Low: p < 0.001; Control vs High: p < 0.001; High vs Low: p = 0.091 Fig. 3a & b).

## 4. DISCUSSION

Together our data indicate that imidacloprid at concentrations commonly occurring in the field^54,59,61^ alter the nervous system of off-target organisms. The density of lip microglomeruli in the honey bee mushroom body decreases as a result of field-relevant, sublethal imidacloprid exposure in adult honey bees. Furthermore, *C. elegans* exposed to field-relevant, sublethal imidacloprid decreased expression of both postsynaptic acetylcholine receptors and presynaptic vesicular acetylcholine transporters. Treatments controlled for differences in lifespan and environmental exposure. In both cases, the model organisms were exposed to chronic field-relevant, sub-lethal concentrations of imidacloprid for about one-sixth of their life expectancies.

Our experimental design, including the model organisms chosen and regions studied, were intentional. The mushroom body of the honey bee is an area critical for learning and memory^62,63^ and has been extensively studied due to its capacity for neuroplasticity, including in response to pesticide exposure^35,37,38^. Alterations in mushroom body synapses have been linked to simple associative learning tasks and more complex foraging behaviors^27,29,32–35^. The complexity of the behavioral repertoire of honey bees are reflected in their brain complexity. To begin addressing the molecular mechanism of the neuronal impact of imidacloprid on cholinergic synapses, we utilized another animal model that has lines expressing fluorescently labeled synaptic structures. While also being important agriculturally, distinctly labeled strains of *C. elegans* exposed to imidacloprid allowed for characterization of the effects on either side of the synapse.

Despite not impacting survival, 7 days of imidacloprid exposure has measurable effects on microglomeruli density (Fig. 1). Imidacloprid exposure irreversibly activates postsynaptic acetylcholine receptors while evading the degradation activity of acetylcholinesterases. This causes decreased activation of the postsynaptic cell after a short period of hyperexcitation, and ultimately convulsion, paralysis, and death of the target organism^64,65^. Imidacloprid has been demonstrated to reduce brain growth and impairs adult learning in honey bees^44,66,67^. Sublethal imidacloprid exposure as a larvae^36^ and post-emergence^38^ alter density of microglomeruli. Peng and Yang^36^ found that larvae fed concentrations of 10 ppb or greater, but not 1 ppb, had decreased microglomeruli density at 20 days post-emergence. Cabirol et al.^38^, as in our study, caged bees following emergence and fed them imidacloprid for 7 days. Interestingly, they found that imidacloprid prevented synaptic pruning, as evidenced by increased microglomeruli density. The doses used, 1 ppb and 5 ppb, were lower than the lowest dose utilized in this study (10 ppb) and lower than the effective dose in Peng and Yang^36^. Interestingly, a concentration of 10 ppb of imidacloprid is required in culture to induce neuronal depolarization^45^. Excitotoxicity requires a threshold to be reached before damage is seen. It is likely that the 10 ppb dose is at the precipice which leads to microglomeruli loss.

The microglomeruli studied here are synaptic structures consisting of the dendrites of postsynaptic Kenyon cells surrounding the presynaptic terminal boutons of projection neurons from the antennal lobes^27^. Changes in the density of the entire microglomeruli structure suggest a dynamic interplay between the pre- and postsynaptic neurons. The nematode data confirm this interplay. *C. elegans*, as natural soil decomposers, are off-target organisms for this pesticide. Thus, they are exposed to concentrations of imidacloprid in their natural habitat. Previous data in the literature finds that although toxic doses of imidacloprid disrupts growth, locomotion and fertility, mortality was unaffected^42^. We found that sub-lethal exposure of imidacloprid does not have significant impacts on the locomotory capabilities in *C. elegans* (data not shown). Since imidacloprid binds to postsynaptic acetylcholine receptors in target organisms, it was hypothesized that transgenic fluorescently labeled acetylcholine receptors in *C. elegans* (CZ631 strain) would be impacted. Our data show a decrease in expression of postsynaptic acetylcholine receptors (Fig. 2), which is in agreement with previous reports in other organisms^68^. This decreased expression is simultaneous with neurite degeneration which is demonstrated through consistent neurite blebbing in the high concentration exposure experiments.

Fewer studies have examined changes in the synaptic partners of these target neurons. Tavares et al.^69^ found a decrease in quantified fluorescence of presynaptic synapsin following another neonicotinoid pesticide, thiamethoxam. We demonstrate here a decrease in expression of transgenic fluorescently labeled vesicular acetylcholine transporters (LX929 strain) of nematodes (Fig. 3), which further supports the impact on the synaptic partner which imidacloprid does not directly bind. A comparative neuroscience approach to the data suggests that the changes in microglomeruli density are initiated by decreasing acetylcholine receptor expression. Further work is required to determine if this is driven by decreased stimulation of the receptor leading to a downregulation of the expression of ‘replacement’ receptors or if imidacloprid-bound receptors become tagged to be endocytosed from the membrane and degraded. We can also conclude that changes initiated on the postsynaptic membranes are leading to presynaptic alterations. Bi-directional communication between the presynaptic terminal and postsynaptic dendrite is required to maintain a functional synapse. We hypothesize that trophic signals released from the postsynaptic neuron are feeding back to the presynaptic terminal. We hypothesize that imidacloprid-induced calcium influx into the postsynaptic neuron may activate pathways that form the retrograde signaling molecules, such as nitric oxide^70^ or an endocannabinoid^71^. Increased release of these highly conserved retrograde signaling molecules have been linked with axonal pruning^72–74^. Nitric oxide has been linked to vesicular transporter modifications that lead to their down-regulation^75^. Further studies are necessary to dissect the specific signals and molecular cascades involved.

Our results support previous research that imidacloprid exposure can change both the membrane properties and functions of neurons that have nicotinic receptors, as reported in neurotoxicology studies in mice^77^. Disturbances in presynaptic vesicular transporter expression have also been linked to many neurophysiological disorders, including Alzheimer’s Disease^77^. These data are important both in correlating structural changes with molecular alterations following imidacloprid, but also in adding to a potential mechanism of the behavioral and physiological changes due to exposure in the environment. Future studies examining at a finer time scale and further elucidating the additional molecular players are necessary to fully understand how imidacloprid, and likely other neonicotinoids, in the environment are altering animals exposed to them.

## Supplementary Material

Supplementary Files

This is a list of supplementary fi les associated with this preprint. Click to download.

• DataforFigure3.zip

• Dataforfi gure1.zip

• DataforFigure2.zip

## Data Availability

All data generated or analyzed during this study are included in the supplementary information files.
